# Blood‐based gene and co‐expression network levels are associated with AD/MCI diagnosis and cognitive phenotypes

**DOI:** 10.1002/alz.086187

**Published:** 2025-01-09

**Authors:** Xuan Chen, Joseph S. Reddy, Xue Wang, Zachary Quicksall, Thuy Nguyen, Denise A. Reyes, Jonathan Graff‐Radford, Clifford R. Jack, Val J. Lowe, David S. Knopman, Ronald C. Petersen, Kejal Kantarci, Kwangsik Nho, Mariet Allen, Minerva M. Carrasquillo, Andrew J. Saykin, Nilüfer Ertekin‐Taner

**Affiliations:** ^1^ Mayo Clinic, Jacksonville, FL USA; ^2^ Mayo Clinic, Rochester, MN USA; ^3^ Department of Neurology, Mayo Clinic, Rochester, MN USA; ^4^ Department of Radiology, Mayo Clinic, Rochester, MN USA; ^5^ Center for Neuroimaging, Department of Radiology and Imaging Sciences, Indiana University School of Medicine, Indianapolis, IN USA

## Abstract

**Background:**

Alzheimer’s disease (AD) patients have decline in cognitive domains including memory, language, visuospatial, and/or executive function and brain pathology including amyloid‐β and tau deposition, neurodegeneration, and frequent vascular co‐pathologies detectable by neuroimaging and/or cerebrospinal fluid biomarkers. However, molecular disease mechanisms are complex and heterogeneous. It is necessary to develop cost‐effective blood‐based biomarkers reflecting brain molecular perturbations in AD. We identified blood‐based gene and co‐expression network level changes associated with AD/mild cognitive impairment (MCI) diagnosis and AD‐related phenotypes.

**Method:**

We performed differential gene expression and weighted gene co‐expression network analysis, followed by meta‐analysis, using blood transcriptome data of 391 participants from the Mayo Clinic Study of Aging and 654 participants from the Alzheimer's Disease Neuroimaging Initiative. The neuroimaging phenotypes include microhemorrhages, infarcts, amyloid burden, hippocampal volume, and white matter hyperintensities. The cognitive phenotypes include standardized cognitive subtest scores and composite scores for memory, language, visuospatial, and executive function.

**Result:**

Five out of 18 modules(M) are significantly associated with diagnosis or cognition (FDR‐adjusted p<0.05). M1 and M15 both positively associates with memory, M1 positively associated with language and M15 with visuospatial function. M1 and M15 are enriched in differentially expressed genes (DEGs) associated with language and executive function, respectively. M2 negatively associates with logical memory delayed recall scores(LMDR), memory, executive, and language functions and is enriched in DEGs for these phenotypes. M8 negatively associates with memory, language and executive functions and is enriched in DEGs for memory and language. M12 positively associates with LMDR. M1 and M15 are down‐regulated while M2 and M8 are up‐regulated in AD/MCI patients. Cell‐type enrichment analysis showed M2 is enriched in monocytes and neutrophils; M8 in monocytes; M15 in B cells (FDR <0.05). Gene ontology terms enriched in these modules indicated broad consistency with their cell types.

**Conclusion:**

We identified five modules significantly associated with AD/MCI or cognitive phenotypes using blood transcriptome data. These findings nominate blood transcriptome changes and their enriched biological processes as potential pathomechanisms in cognitive decline and AD/MCI development. We aim to investigate these blood transcripts as potential biomarkers for AD or AD‐related phenotypes and therapeutic targets through additional replication and experimental validation studies.

